# Maternal autoimmune disease and offspring risk of haematological malignancies: a case–control study

**DOI:** 10.1016/j.eclinm.2024.102794

**Published:** 2024-08-30

**Authors:** Shu-Ning Liu, Meng-Che Wu, Wei-Szu Lin, Ching-Heng Lin, James Cheng-Chung Wei

**Affiliations:** a Children's Medical Center, Taichung Veterans General Hospital, Taichung, Taiwan; b Department of Post-Baccalaureate Medicine, College of Medicine, National Chung Hsing University, Taichung, Taiwan; c School of Medicine, Chung Shan Medical University, Taichung, Taiwan; d Center for Pediatric Inflammatory Bowel Disease, MassGeneral Hospital for Children, Boston, MA, USA; e Department of Medical Research, Taichung Veterans General Hospital, Taichung, Taiwan; f Department of Nursing, Chung Shan Medical University, Taichung, Taiwan; g Department of Allergy, Immunology & Rheumatology, Chung Shan Medical University Hospital, Taichung, Taiwan; h Institute of Medicine, Chung Shan Medical University, Taichung, Taiwan; i Graduate Institute of Integrated Medicine, China Medical University, Taichung, Taiwan

**Keywords:** Autoimmune disease, Rheumatologic disease, Haematologic malignancy, Leukaemia

## Abstract

**Background:**

Autoimmune diseases are known to be associated with an increased risk of cancer. Whether maternal immune dysregulation can have an impact on the development of haematological malignancies in offspring remains uncertain. Therefore, we explored the association between offspring risk of haematological malignancies and maternal autoimmune disease using a real-world nationwide population-based study.

**Methods:**

In this case–control study, we identified 2172 children with haematological malignancies between 2004 and 2019 from Taiwan's National Health Insurance program and compared them with population-based controls without haematologic malignancies, who were matched with each individual at a ratio of 1:4. The medical information of the autoimmune mothers were obtained from the Taiwan Maternal and Child Health Database. Conditional logistic regression was used to estimate the odds ratio for haematologic malignancy in offspring. Furthermore, subgroup and stratified analyses were conducted.

**Findings:**

Among the rheumatologic diseases in our study, Crohn's disease was the most common disease both in the haematological malignancy group (1.1%) and the control group (0.9%). In multivariable analysis, the odds ratio for haematological malignancy in offspring with maternal autoimmune diseases was 1.2 (95% confidence interval [CI] 0.91–1.58). The overall risk of haematologic malignancy was not significantly higher when adjusted for specific risk factors, including neonatal age, maternal age, family income, urbanization, maternal occupation, birth weight, or maternal comorbidity, except for prematurity. When comparing different autoimmune diseases among haematological malignancies and the control group, maternal psoriatic arthritis/psoriasis had the highest adjusted overall risk for haematological malignancies (adjusted OR 2.11, CI 0.89–5), followed by ankylosing spondylitis (adjusted OR 1.45, CI 0.7–3), autoimmune thyroiditis (OR 1.26, CI 0.57–2.81), systemic lupus erythematosus (OR 1.21, CI 0.48–3.02), Crohn's disease (OR 1.19, CI 0.75–1.9), and Sjogren's syndrome (OR 1.18, CI 0.65–2.15), but no significance was reached in these analyses. Multivariable analysis of risk factors associated with haematological malignancy subtypes was done. It showed no associations between maternal autoimmune disease and childhood haematological malignancies.

**Interpretation:**

We found no significant relationship between maternal autoimmune disease and childhood haematological malignancies. The influence of maternal immune dysregulation on the next generation with respect to haematological malignancies development may be limited.

**Funding:**

There was no funding source for this study.

**Research in context**

**Evidence before this study**

During our literature review, we searched on PubMed and Medline with the term “maternal autoimmune disease”, “autoimmune disease”, “childhood malignancy”, “childhood Leukaemia” for research done between 1970 and 2023 on Jul.01.2023. Those irrelevant of the relationship between maternal autoimmune disease and childhood malignancy were excluded. These previous studies results have been conflicting and have been limited by sample size, imprecision and limited definition. Studies on large populations with accurate and complete data identification are needed to confirm the aforementioned hypothesis.

**Added value of this study**

In our study, the maternal autoimmune diseases were not associated with an increased risk for cancer in offspring (adjusted OR 1.2, 95% CI 0.91–1.58) when compared to the offspring of mothers with no autoimmune disease. Maternal psoriatic arthritis/psoriasis had the highest adjusted overall risk for haematologic malignancy (adjusted OR 2.11, 95% CI 0.89–5), followed by ankylosing spondylitis and autoimmune thyroiditis, but no significance was reached. Our study utilized data collected from a large database that covers 99% of the Taiwanese population, as well as the Taiwan Maternal and Child Health Database, ensuring accurate linkage between children and their mothers. Besides, our study had a larger population size and encompassed a wide range of autoimmune diseases and haematologic malignancies, allowing for a more comprehensive analysis.

**Implications of all the available evidence**

In this study, our findings indicate that there appears to be a limited influence of maternal immune dysfunction on the next generation. The well-controlled management of maternal autoimmune diseases during pregnancy could potentially explain their minimal impact on the fetus.

## Introduction

Autoimmune disorders occur when the immune system mistakenly attacks the host's tissue due to failure in distinguishing between host and foreign antigens. They are prevalent in women, with a ratio of up to 9:1 versus men, often manifest between the ages of 15 and 40, i.e., the reproductive age.^1^

Lymphohematopoietic cancers, including acute lymphoblastic leukaemia (ALL), myeloid leukaemia (AML), Hodgkin and non-Hodgkin lymphomas, account for approximately 40% of pediatric malignancies. They are among the leading non-accidental cause of death among the pediatric population in developed countries and cause significant mortality and morbidity. Other than exposure to radiation, genetic abnormalities, certain viral infections or chemotherapeutic drugs, the etiology of childhood haematologic malignancy remains unknown.^2^

Studies on the relationship between autoimmune disorders and risk of malignancy in the same individual are abundant.^3^, ^4^, ^5^, ^6^ However, autoimmune disorders in the relatives of patients with haematologic malignancies were less studied. There have been some studies focusing on possible familial links between autoimmune diseases and haematopoietic malignancies with inconsistent results. Whether maternal immune dysregulation has an impact on malignancy development in offspring has long been a topic of interest in the literature. Some showed increased prevalence of autoimmune disorder in relatives of patients with ALL,^7^, ^8^, ^9^ or relationship between maternal multiple sclerosis and childhood leukaemia.^10^ Moreover, a case–control study in France found an association between childhood leukaemia and autoimmune disease in first/second degree relatives, especially in autoimmune thyroid diseases (Grave's and Hashimoto's disease).^7^ A cohort study conducted in Denmark indicated an increased risk of childhood leukaemia and lymphoma among children with parental autoimmune disease.^9^

There were also several studies that reached conclusions that differed from the above-mentioned analysis. Two case–control studies in the United States and United Kingdom found no relationship between childhood cancers and maternal autoimmune diseases.^11^^,^^12^ Three studies using country-wide health registers in Finland, Sweden, United States, and Canada reached the same conclusion.^13^, ^14^, ^15^, ^16^ A large cohort that included 76,527 patients from Swedish nationwide registers found no relationship between patients with rheumatoid arthritis and malignancy risk in their offspring, but only one autoimmune disease was analysed in the study.^14^ Heather et al. in the United States conducted a case–control study using Children's Cancer Group institutions.^11^ Mckinney et al. in the United Kingdom also conducted a similar study in an attempt to analyse a wide range of potential risk factors of childhood leukaemia or lymphoma.^12^

These previous studies results have been conflicting and have been limited by sample size, imprecision and limited definition. Studies on large populations with accurate and complete data identification are needed to confirm the aforementioned hypothesis. Therefore, in this nationwide case–control study in Taiwan, we aimed to investigate the role of maternal autoimmune disease and any associations with childhood haematologic malignancies.

## Methods

### Data source

The study population was extracted from Taiwan's National Health Insurance (NHI) program, which was established by the National Health Insurance Administration in 1995 and currently covers up to 99% of Taiwan's population of 23 million.^17^ This national insurance program covers outpatient medical expenses, inpatient and emergency care, dental services, and prescription drugs for NHI beneficiaries. The Bureau of National Health Insurance regularly reviews NHI data to ensure accuracy and integrity. The National Health Insurance Research Database (NHIRD) was created by the National Health Research Institutes, which is funded by the Bureau of National Health Insurance. The database comprises data provided by the NHI program and is used for medical research and statistical analysis.

### Participant selection

We collected data from NHIRD for participant selection. This database was representative of the whole insured population of Taiwan. The study population comprised 2,952,666 newborns born between 2004 and 2019. Haematologic malignancy was identified by searching for the relevant International Classification of Diseases (ICD) codes. We identified 2172 children with haematologic malignancy between 2004 and 2019, including children diagnosed with leukaemia (ICD Ninth Revision, Clinical Modification code 204–208, ICD-10 CM C91-95), lymphosarcoma and reticulosarcoma, and other specified malignant tumours of lymphatic tissue (ICD-9 CM code 200-202, ICD-10 CM C81-86, C88). Taiwan's NHI provides coverage for nearly all of the population, so the number of childhood haematologic malignancy cases in the database should be close to the number of real-world cases.^18^ Our study involved children born between 2004 and 2019, and the follow-up duration ranged from 1 to 16 years.

For each patient with haematologic malignancy, four controls were selected. These controls were matched based on three criteria: maternal age, gender, and the age on index date. The index date represents the specific day when the individual received a diagnosis of a haematologic malignancy. These information was extracted from the Longitudinal Health Insurance Database, a subset of the NHIRD. The maternal medical history of these children was also collected from the same database. Both the diagnosis of haematologic malignancy and autoimmune disease was defined by at least 1 hospital admission and 3 outpatient department visits. The children's maternal age, gestation age, and birth weight were gathered from birth databases. Family income, urbanization, and maternal occupation were obtained from data from the Taiwan National Health Insurance program. Because all of the data were obtained from records in the NHIRD, the participation rate of the study participants was 100%. This study was approved by the Institutional Review Board of Taichung Veterans General Hospital (IRB number: CE17178A-5).

### Data collection

#### Maternal autoimmune disease

Autoimmune diseases were identified by searching for the relevant ICD codes in the NHIRD, including systemic lupus erythematosus (SLE) (ICD-9 CM 710, ICD-10 CM M32), rheumatoid arthritis (ICD-9 CM 714, ICD-10 CM M06.9), Sjogren's syndrome (ICD-9 CM 710.2, ICD-10 CM M35), ankylosing spondylitis (ICD-9 CM 720, ICD-10 CM M45), psoriatic arthritis (ICD-9 CM 696, ICD-10 CM L40.52), autoimmune thyroiditis (ICD-9 CM 245.2, ICD-10 CM E06.3), and Crohn's disease (ICD-9 CM 555.9, ICD-10 CM K50.9). The confirmation of maternal autoimmune diseases were conducted using 2 NHIRD files: 1) Ambulatory Care Expenditures by Visits; and 2) Inpatient Expenditures by Admission. The diagnosis was defined as at least 1 hospital admission or 3 outpatient department visits.

#### Covariates

We identified potential factors associated with childhood malignancy as confounders, included family income, urbanization, and maternal occupation through literature review.^14^^,^^19^, ^20^, ^21^, ^22^, ^23^ We included family income, urbanization, and maternal occupation as potential confounders. The following methods were used for data collection. Income-related insurance amount of each patient's parent (father or mother) was considered to be representative of family income, with 18,780 New Taiwan Dollars being the lowest income-related insurance amount. City of residency was used to determine the level of urbanization.

#### Statistical analysis

Chi-squared tests were used in the comparisons of two groups between family income, urbanization, maternal occupation, gestation in completed weeks, birth weight, maternal comorbidity, and the rheumatologic section, including systemic lupus erythematosus, rheumatoid arthritis, Sjogren's syndrome, ankylosing spondylitis, autoimmune thyroiditis and Crohn's disease. In the comparison of psoriatic arthritis/psoriasis, the expected cell counts are less than 5. Fisher's exact test was used instead. P values were 2-sided, and statistical significance was set at P < 0.05. Conditional logistic regression was performed to generate odds ratios (OR) and 95% confidence intervals (CIs) for estimating the risk of childhood haematologic malignancy associated with maternal autoimmune disease. Analysis of overall risk for childhood haematologic malignancy was done with different kinds of maternal autoimmune diseases as variables. Multivariable analysis of different haematologic malignancy groups was done adjusted for different variables. Continual variables (neonatal age, maternal age, family income, gestational age and birth weight) and categorical variables (gender, maternal occupation, urbanization level, and maternal comorbidity) were calculated separately.^24^, ^25^, ^26^ For continual variables, multivariable fractional polynomials were applied for analysis. OR estimates with 95% CIs were also provided. Analyses were performed using SAS Enterprise Guide statistical software, version 9.4 (SAS Institute).

### Ethics

The studies involving human participants were reviewed and approved by the Institutional Review Board of Taichung Veterans General Hospital (IRB number: CE17178A-5). Written informed consent for participation was not required for this study in accordance with the national legislation and the institutional requirements.

### Informed consent statement

Patient consent was waived because all the data from NHIRD were delinked from private information before released to the researchers.

### Role of the funding source

All authors have confirmed that they had full access to all the data in the study. All authors have accepted responsibility for the decision to submit for publication There was no funding source for this study.

## Results

**Fig. 1:**
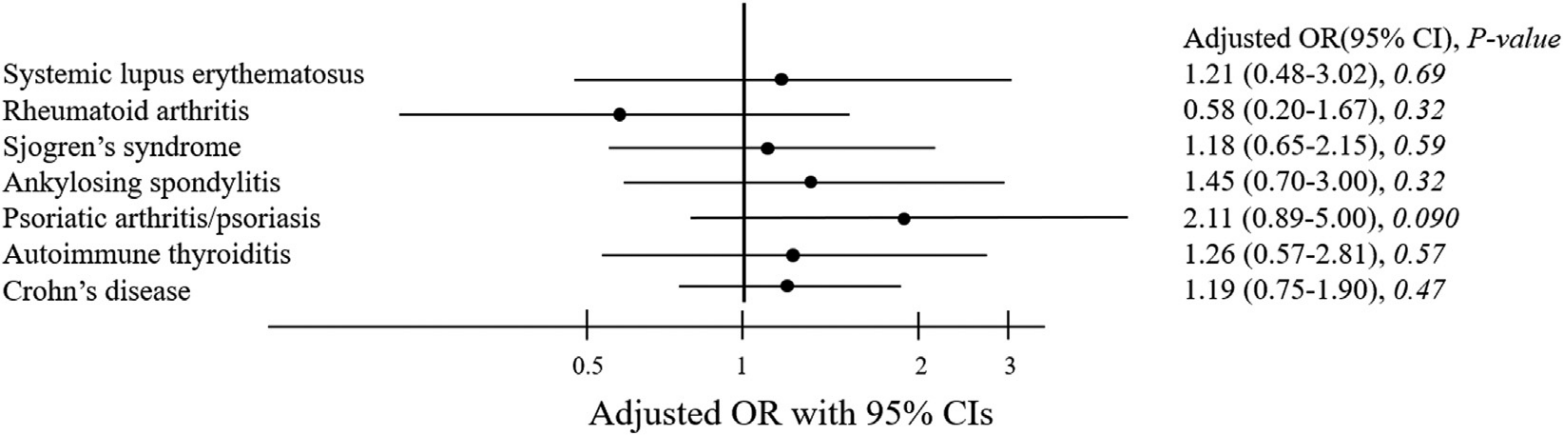
Different maternal autoimmune diseases and risk of the haematologic malignancy in offspring. Overall risk for childhood haematologic malignancy was analysed with different kinds of maternal autoimmune diseases as variables. No significance was observed in these analysis. OR: odds ratios, CI: confidence intervals.

The flow chart of cases collection is shown in Appendix 1. We enrolled 2172 patients with haematologic malignancy (1240 boys [57.1%] and 932 girls [42.9%]) and 8688 matched individuals without haematologic malignancy (4960 boys [57.1%] and 3728 girls [42.8%]) from 2,952,622 children in the database. For the characteristics of the cases and the control group, see Table 1. The control group had significantly more cases with gestational age more than 37 weeks compared with haematologic malignancy group. Crohn's disease was the most common disease both in the haematologic malignancy group and the control group, followed by Sjogren's syndrome and ankylosing spondylitis. In the multivariable analysis (Table 2), the association between maternal autoimmune diseases and an increased risk for cancer in offspring (adjusted OR 1.2, 95% CI 0.91–1.58) was not observed when compared the offspring of mothers with no autoimmune disease, except for younger neonatal age (adjusted OR 0.31, 95% CI 0.23–0.41, P-value <0.0001) gestation age less than 37 weeks (adjusted OR 0.19, 95% CI 0.07–0.51, P-value: 0.001) and relatively low birth weight (adjusted OR 1 CI 1–1, P-value: 0.047). When comparing different autoimmune diseases between the haematologic malignancy and control groups, maternal psoriatic arthritis/psoriasis had the highest adjusted overall risk for haematologic malignancy (adjusted OR 2.11, 95% CI 0.89–5), followed by ankylosing spondylitis (adjusted OR 1.45, CI 0.7–3), autoimmune thyroiditis (OR 1.26, CI 0.57–2.81), systemic lupus erythematosus (OR 1.21, CI 0.48–3.02), Crohn's disease (OR 1.19, CI 0.75–1.9), and Sjogren's syndrome (OR 1.18, CI 0.65–2.15), but no significance was observed in these analysis (Fig. 1). Multivariable analysis of risk factors associated with haematologic malignancy subtypes were done and shown in Table 3. The association between any maternal autoimmune disease and childhood haematologic malignancies was not evident in this analysis. The same conclusion was observed among acute lymphocytic leukaemia, acute myeloid leukaemia, or lymphoma.

**Table 1:** Characteristics of study subjects.

Characteristic	Control group (n = 8688) n (%)	Hematologic malignancy group (n = 2172) n (%)	Total n	P-value
**Neonatal age(years), Median (IQR, Q1-Q3)**	3.70 (4.92, 1.95–6.87)	3.6 (4.58,1.79–6.37)		
**Gender**				
Female	3728 (42.9)	932 (42.9)	4660	
Male	4960 (57.1)	1240 (57.1)	6200	
**Maternal age(years), Mean** ± **SD**	30.16 ± 4.87	30.15 ± 4.99		
**Family income, Median (IQR, Q1-Q3)**	21,900 (16,500, 18,300–34,800)	24,000 (20,827, 19,273–40,100)		0.61
**Urbanization**				0.60
Urban	5160 (59.4)	1264 (58.2)	6424	
Suburban	1190 (13.7)	306 (14.1)	1496	
Rural	2338 (26.9)	602 (27.7)	2940	
**Maternal occupation**				0.12
White + semi-white	5778 (66.5)	1426 (65.7)	7204	
Semi-blue + blue	1651 (19)	452 (20.8)	2103	
Others	1259 (14.5)	294 (13.5)	1553	
**Gestation in completed weeks, Mean** ± **SD**	38.33 ± 1.55	38.24 ± 1.65		0.040
**Birth weight(g), Mean** ± **SD**	3091.9 ± 436.5	3099.9 ± 439.4		0.45
**Maternal comorbidity**				
Diabetes mellitus	228 (2.6)	55 (2.5)	283	0.81
Hypertension	144 (1.7)	35 (1.6)	179	0.88
**Rheumatologic**				
**All**	224 (2.6)	68 (3.1)	292	0.16
Systemic lupus erythematosis	20 (0.2)	6 (0.3)	26	0.67
Rheumatoid arthritis	27 (0.3)	4 (0.2)	31	0.32
Sjogren's syndrome	47 (0.5)	14 (0.6)	61	0.56
Ankylosing spondylitis	27 (0.3)	10 (0.5)	37	0.28
Psoriatic arthritis/psoriasis^a^	15 (0.2)	8 (0.4)	23	0.11^b^
Autoimmune thyroiditis	25 (0.3)	8 (0.4)	33	0.54
Crohn's disease	79 (0.9)	23 (1.1)	102	0.52

.

a The characteristics of both groups were compared with Chi square test, except for ‘Psoriatic arthritis/psoriasis’.

b Fisher's exact test was used due to the expected cell counts are less than 5.

**Table 2:** Multivariable analysis of factors associated with hematologic malignancy.

Variables	Model			
	Adjusted OR	95% CI		P-value
**Rheumatologic**				
All	1.20	0.91	1.58	0.208
**1/(Neonatal age)**^**2**^**+ log(Neonatal age)1/(Neonatal age)**^**2**^^a^	0.31	0.23	0.41	<0.0001
**Gender**				
Female	1.00			
Male	0.98	0.89	1.08	0.712
**(Maternal age)**^**3**^^a^	1.00	1.00	1.00	0.981
**1/(Family income)**^a^	1.35	0.14	13.00	0.795
**Urbanization**				
Urban	1.00			
Suburban	1.06	0.92	1.22	0.445
Rural	1.05	0.94	1.17	0.394
**Maternal occupation**				
White + semi-white	1.00			
Semi-blue + blue	1.12	0.99	1.26	0.070
Others	0.94	0.82	1.08	0.391
**(Gestation in completed weeks)**^**2**^^a^	1.00	1.00	1.00	0.019
**(Birth weight)**^**3**^^a^	1.00	1.00	1.00	0.047
**Maternal comorbidity**				
Diabetes mellitus	0.94	0.69	1.28	0.681
Hypertension	0.95	0.65	1.39	0.781
Hyperlipidemia	1.14	0.81	1.62	0.450

OR: odds ratios, CI: confidence intervals.

a Continual variables were calculated separately from categorical variables.

**Table 3:** Multivariable analysis of factors associated with hematologic malignancy.

Variable	Case	N	Adjusted OR	95% CI		P-value
Non-Haematologic Malignancy group	224	8688	1.00			
Haematologic Malignancy group						
Acute lymphocytic leukaemia	37	1112	1.26	0.88	1.80	0.20
Acute myeloid leukaemia	8	290	1.10	0.53	2.26	0.80
Leukaemia (others)	6	144	1.46	0.63	3.40	0.38
Hodgkin's lymphoma	1	45	1.21	0.16	9.11	0.86
Non-Hodgkin's lymphoma	16	571	1.15	0.15	8.67	0.90

Model adjusted for neonatal age, gender, maternal age, family income, urbanization, maternal occupation, gestation in completed weeks, birth weight, maternal comorbidity.

OR: odds ratios, CI: confidence intervals.

## Discussion

This study, one of the largest case–control studies on maternal history and childhood haematologic malignancy so far to our knowledge, found no increased risk of haematologic malignancy in children born to mothers with autoimmune diseases, despite the high prevalence of these diseases among women of reproductive age. The analysis considered various risk factors, such as neonatal age, maternal age, family income, urbanization, maternal occupation, gestation period, birth weight, and maternal comorbidity, but found no significant associations. Additionally, specific types of haematologic malignancies, including ALL, AML, and lymphomas, did not show heightened risks. Autoimmune diseases predominantly affect women^27^, ^28^, ^29^ and immune dysfunction has been linked to tumour development.^30^, ^31^, ^32^, ^33^ Therefore, further investigation is needed to fully understand the potential effects of autoimmune diseases on offspring.

We conducted a literature review and found four early studies that investigated the association between familial autoimmune disease and childhood leukaemia. Three of these studies reported positive results. Till et al. (1978) reported that parents of children with acute lymphoblastic leukaemia had a significantly higher prevalence of autoimmune diseases. However, the study had a small sample size of less than 100 cases in the analysis.^7^ Buckley et al. (1989) found a significant excess of multiple sclerosis among mothers of children with ALL.^10^ Perillat et al. (2003) observed an increased risk of autoimmune disease in first- or second-degree relatives of children with ALL, as well as an association between certain thyroid diseases (Graves' disease, hyperthyroidism, and Hashimoto's disease) and ALL.^8^ McKinney et al. (1987) found that prenatal factors were less influential in the development of childhood leukaemia and lymphoma.^12^ Nationwide health register databases were used to conduct similar studies in Denmark, Sweden, and Finland.^13^, ^14^, ^15^, ^16^ One study, by L. Mellemkjaer et al. (from Denmark), found a 1.6-fold increased risk of lymphomas among children with parental autoimmune disease.^9^ However, no excess risk was observed for ALL in this study. Other studies using population-based registers in Sweden and Finland did not find a significant association between maternal autoimmune diseases and childhood leukaemia, but there was a slightly increased risk of malignant lymphomas and Hodgkin's lymphoma observed in some cases.^13^^,^^14^ Finally, Linabery, Amy M. et al., in 2014 concluded that family history of immune dysregulation was associated with childhood Hodgkin lymphoma and EBV infection.^34^

It is important to note that the earlier studies relied on questionnaires for data collection, which may have introduced recall bias. Additionally, the definition of autoimmune diseases in these studies was limited to specific types of disorders. Furthermore, these studies focused primarily on acute lymphoblastic leukaemia, excluding other important childhood haematologic malignancies, such as myeloid leukaemia, Hodgkin lymphoma, and non-Hodgkin lymphoma.

In this study, our findings indicate that there appears to be a limited influence of maternal immune dysfunction on the next generation. The well-controlled management of maternal autoimmune diseases during pregnancy could potentially explain their minimal impact on the fetus. Our study, in comparison to previous research on the same topic, utilised data collected from the NHIRD, a large database that covers 99% of the Taiwanese population, as well as the Taiwan Maternal and Child Health Database, ensuring accurate linkage between children and their mothers. These systems have been in use for over 20 years and undergo regular reviews, thus minimizing the potential for recall errors or bias in medical records. In contrast, previous studies relied on data gathered from parental recall, which may introduce speculation and lead to false–positive associations. Additionally, our study had a larger population size and encompassed a wide range of autoimmune diseases and haematologic malignancies, allowing for a more comprehensive analysis. Previous studies were often limited to specific autoimmune diseases or focused on only one type of childhood cancer, which may have contributed to the differing results observed compared to our study.

There were several limitations in our study. The wide confidence intervals observed in our study reflect uncertainty regarding the estimated effects of the variables under investigation. Although we adjusted for a variety of relevant factors and comorbidities to reduce the confounders, unmeasured factors might have biased our results. These intervals indicate that the true value of the parameter being estimated could vary considerably within the stated range. We focused on childhood haematologic malignancies, so further investigation is still needed to assess the role of maternal autoimmune disease in the development of non-haematologic malignancies. Additionally, the potential impact of maternal autoimmune disease on malignancy development later in life remains unknown, as our study solely focused on the pediatric age range.

## Contributors

Shu-Ning Liu was responsible for study design, analysis and interpretation of data, original draft preparation, review and editing. Meng-Che Wu was responsible for study design, analysis and interpretation of data, original draft preparation, review and editing. Wei-Szu Lin was responsible for accessing, verifying the underlying data, analysis and interpretation of data. Ching-Heng Lin PhD was responsible for accessing, verifying the underlying data, analysis, interpretation of data, review and editing. James Cheng-Chung Wei MD PhD was responsible for study design, analysis and interpretation of data, review and editing. All authors have full access to all the data in the study and accept responsibility for the decision to submit for publication.

## Data sharing statement

The data mentioned in the manuscript could be accessed through the National Health Insurance Research Database provided by Taiwan national health insurance program.

## Declaration of interests

The authors have no conflicts of interest to declare.
