# Supplementary figures and images for: Maternal autoimmune disease and offspring risk of haematological malignancies: a case–control study
Source: eClinicalMedicine. 2024 Aug 30;75:102794. doi: 10.1016/j.eclinm.2024.102794 (PMC11402410; doi:10.1016/j.eclinm.2024.102794)

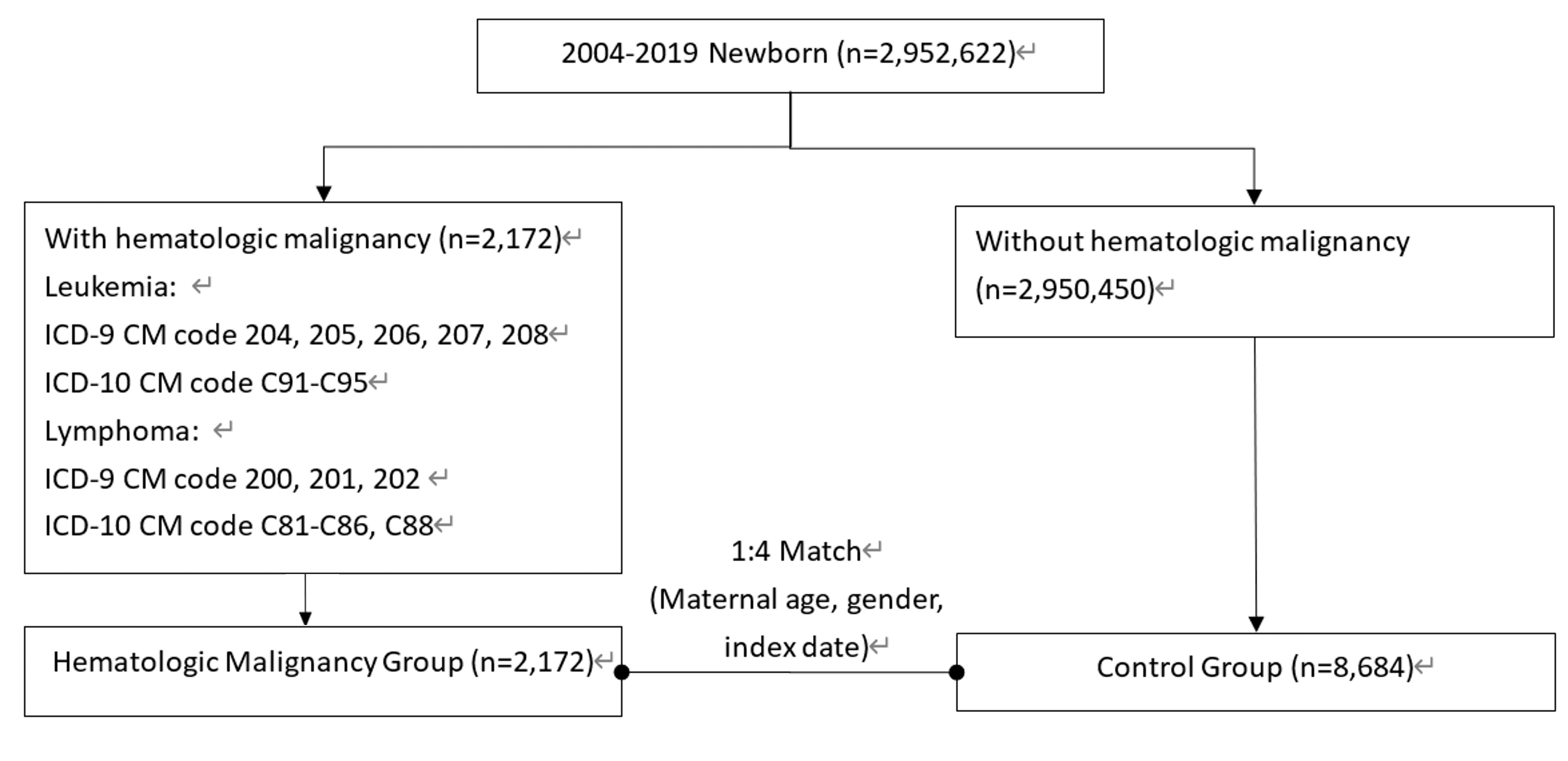


Appendix

Study flowchart.

Supplement: Appendix [file mmc1.docx]
